# Supplementary material for: Survival Comparison of Different Operation Types for Middle Bile Duct Cancer: Bile Duct Resection versus Pancreaticoduodenectomy Considering Complications and Adjuvant Treatment Effects
Source: Cancers (Basel). 2024 Jan 10;16(2):297. doi: 10.3390/cancers16020297 (PMC10814212; doi:10.3390/cancers16020297)
Supplement: Supplementary file 1 [file cancers-16-00297-s001.zip › cancers-2764566-supplementary.pdf]

Supplementary Materials: Survival Outcomes for Different Operation Types in the Era of Modern Chemotherapy for Middle Bile Duct Cancer: Bile Duct Resection versus Pancreaticoduodenectomy Considering Complications and Adjuvant Treatment Effects

Soo Yeun Lim, Hani Jassim Alramadhan, Hye Jeong Jeong, Hochang Chae, Hyeong Seok Kim, So Jeong Yoon, Sang Hyun Shin, In Woong Han, Jin Seok Heo and Hongbeom Kim

Table S1. Clinical Course of R1 Patients (*n* = 6).

No.	Frozen biopsy (intraoperative)	Frozen biopsy (permanent)	Reason for R1	Final Stage	Adjuvant Treatment
1	Carcinoma	Carcinoma	Patients condition, PHx	IIB	RTx
2	Carcinoma	Carcinoma	Patients condition, PHx	IIIB	X
3	Atypical cell	Carcinoma	Changed Frozen biopsy result	IIA	X
4	Highly Atypical cell	Carcinoma	Changed Frozen biopsy result	IIB	X
5	High grade dysplasia	Carcinoma	Changed Frozen biopsy result	I	X
6	High grade dysplasia	Carcinoma	Changed Frozen biopsy result	IIB	RTx

PHx, Past history; RTx, radiotherapy.

Table S2. Prognostic Factors for Disease-Free Survival in Patients with Middle Bile Duct Cancer (*n* = 520).

Variable		Patients (n)	5Y DFS (%)	Univariate analysis			Multivariate analysis		
				HR	95% CI	*p*-value	HR	95% CI	*p*-value
Sex	male / female	342/178	36.5/40.5	0.850	0.679-1.064	0.157			
Age	≤ 65 / > 65	229/291	47.5/29.7	1.559	1.251-1.944	<0.001	1.616	1.293-2.020	<0.001
BMI	≤ 25 / > 25	378/142	36.2/41.9	0.841	0.657-1.076	0.167			
ASA score	I	82	35.8			0.256			
	II	395	40.3	0.944	0.709-1.257	0.694			
	III/IV	43	19.0	1.276	0.834-1.951	0.262			
Preop CA19-9	≤ 35 / > 35	265/255	44.5/31.1	1.538	1.243-1.902	<0.001	1.335	1.076-1.657	0.009
T-stage	T1&2/T3&4	448/72	41.1/17.2	2.001	1.517-2.637	<0.001	1.499	1.124-1.998	0.006
N-stage	N(-)/N(+)	359/161	45.8/20.2	2.120	1.702-2.640	<0.001	1.965	1.558-2.478	<0.001
Operation	BDR / PD	131/389	34.4/39.0	0.970	0.762-1.235	0.803			
Resection margin	R0 / R1	514/6	38.4/0	1.858	0.827-4.172	0.127			
Complications	no / yes	389/131	39.6/32.3	1.111	0.871-1.418	0.395			
Adjuvant treatment	no / yes	403/117	39.3/32.9	1.179	0.918-1.514	0.198			

Major complication indicated Clavien-Dindo grade ≥ 3. PD/PPPD, pancreaticoduodenectomy/pylorus-preserving pancreaticoduodenectomy; BDR, Bile duct resection.
